# A database of calculated solution parameters for the AlphaFold predicted protein structures

**DOI:** 10.1038/s41598-022-10607-z

**Published:** 2022-05-05

**Authors:** Emre Brookes, Mattia Rocco

**Affiliations:** 1 Department of Chemistry and Biochemistry, The University of Montana, 32 Campus Dr, Missoula, MT 59812 USA; 2 Proteomica e Spettrometria di Massa, IRCCS Ospedale Policlinico San Martino, Largo R. Benzi 10, 16132 Genova, Italy

**Keywords:** Protein databases, Computational biophysics, SAXS, Molecular modelling

## Abstract

Recent spectacular advances by AI programs in 3D structure predictions from protein sequences have revolutionized the field in terms of accuracy and speed. The resulting “folding frenzy” has already produced predicted protein structure databases for the entire human and other organisms’ proteomes. However, rapidly ascertaining a predicted structure’s reliability based on measured properties in solution should be considered. Shape-sensitive hydrodynamic parameters such as the diffusion and sedimentation coefficients (${D_{t(20,w)}^{0}}$, ${s_{{\left( {{20},w} \right)}}^{{0}} }$) and the intrinsic viscosity ([*η*]) can provide a rapid assessment of the overall structure likeliness, and SAXS would yield the structure-related pair-wise distance distribution function *p*(*r*) vs. *r*. Using the extensively validated UltraScan SOlution MOdeler (US-SOMO) suite, a database was implemented calculating from AlphaFold structures the corresponding ${D_{t(20,w)}^{0}}$, ${s_{{\left( {{20},w} \right)}}^{{0}} }$, [*η*], *p*(*r*) vs. *r*, and other parameters. Circular dichroism spectra were computed using the SESCA program. Some of AlphaFold’s drawbacks were mitigated, such as generating whenever possible a protein’s mature form. Others, like the AlphaFold direct applicability to single-chain structures only, the absence of prosthetic groups, or flexibility issues, are discussed. Overall, this implementation of the US-SOMO-AF database should already aid in rapidly evaluating the consistency in solution of a relevant portion of AlphaFold predicted protein structures.

## Introduction

The Anfinsen dogma, that protein sequences dictates their three-dimensional (3D) structure, was postulated nearly 50 years ago^1^. It set in motion a quest to find methods to reliably and accurately predict 3D protein structures from their sequence, which became even more important with the full sequencing of the human and other genomes (see https://www.ncbi.nlm.nih.gov/genome). Recent spectacular advances in the 3D structure prediction from protein sequences by Artificial Intelligence (AI) programs such as AlphaFold (AF) and RoseTTAfold appear to have revolutionized the field in terms of accuracy and speed^2,3^. Boosted by their success in predicting structures to near (and sometimes even better than) crystallographic accuracy, the AlphaFold consortium (https://alphafold.ebi.ac.uk) has already made publicly available a series of databases of predicted protein structures first for the entire human and several other organisms proteomes^4^, and more recently for the entire UniProt database of curated sequences^5^ (https://www.uniprot.org).

However, these AI programs have not tackled the folding issue from a thermodynamic/mechanistic approach, but rather by combining many different observations in a deep learning process^6,7^. Apart from simple cases of highly homologous sequences, or clearly recognized folding classes, to reasonably rapidly ascertain the degree of confidence of a predicted structure based on a few measured properties in solution we believe should become a necessary step. For instance, besides known occurrences of multi-chain proteins, determining a molecular mass *M* in solution can immediately verify the protein oligomerization state and prompt for the need of further modeling. On a different level, circular dichroism (CD) spectroscopy, possible on very small sample amounts^8^, would permit a rapid check of the actual secondary structure content of a predicted 3D structure.

Particularly useful for known single-chain proteins in the AF databases, shape-sensitive hydrodynamic parameters such as the translational diffusion and sedimentation coefficients (${D_{t(20,w)}^{0}}$, ${s_{{\left( {{20},w} \right)}}^{{0}} }$) and the intrinsic viscosity ([*η*]), could provide a robust assessment of the overall fold likeliness. These measurements, requiring little material and with a reasonably quick turnaround, are usually accessible in most research endeavors, especially in core facilities where analytical ultracentrifugation^9,10^, multi-angle static and dynamic light scattering (MALS and DLS) coupled to size-exclusion chromatography (SEC)^11,12^ or directly on plate readers^13^, and SEC-coupled differential viscosimetry^14,15^, can often be found. On another level, small-angle X-ray scattering (SAXS) measurements can provide the rms radius of gyration *R*_*g*_ and the pair-wise distance distribution function *p*(*r*) vs. *r*^16–18^. Notably, several synchrotron beamlines offer on-line SEC-SAXS (e.g., Table 11.1 in Ref.^19^), some accepting mailed-in samples for this set-up (e.g., https://www.diamond.ac.uk/Users/Support-for-European-Access-to-Life-Sciences/Applications/Bio-SAXS.html; https://www.embl-hamburg.de/biosaxs/mailin.html; https://www.synchrotron-soleil.fr/en/beamlines/swing; https://bl1231.als.lbl.gov/htsaxs).

Importantly, all these parameters and functions can be calculated, with varying degree of accuracy, from 3D structures. Among the CD spectra computational methods available, we have chosen SESCA, which appears to offer very accurate results for a wide variety of structures^20^. The computation of the hydrodynamic parameters from atomic level structures is a mature field, with several approaches and corresponding software available, and with an average accuracy comparable to that of the experimentally determined parameters, 2–4%^21,22^. For the hydrodynamic and the *p*(*r*) vs. *r* distribution calculations, we have employed the extensively validated UltraScan SOlution MOdeler (US-SOMO) public domain suite^23–25^.

This effort has allowed us to produce and make publicly available, from the AlphaFold released predicted protein structures databases, the comprehensive US-SOMO-AF database presented here, containing the corresponding calculated *M*, ${D_{t(20,w)}^{0}}$, ${s_{{\left( {{20},w} \right)}}^{{0}} }$, [*η*], *p*(*r*) vs. *r*, CD spectra, and other ancillary information. Note that the AlphaFold databases were generated from the UniProt sequences without being curated any further. For instance, many proteins are synthesized with either an initiator methionine^26^, a signal peptide^27^, or a transit peptide^28^, which will be post-translationally removed. In addition, some proteins are also further processed by removal of one or more propeptide sequences (see https://www.uniprot.org/help/ptm_processing_section). These modifications will affect the calculated parameters in an inverse proportion to protein size. As the mature form will be nearly always purified and studied, we have by default removed whenever possible the UniProt-identified initiator, signal, and transit peptide residues from the AF structures before performing the hydrodynamic, structural and spectroscopic calculations. For the propeptides, we have instead generated alternate AF structure(s) when they were removed (see Supplementary Methods for details).

Based on the calculated values, some analyses regarding the effectiveness of performing a screening of predicted structures against experimental parameters are presented. Advantages, drawbacks, and potential improvements are then discussed.

## Results

### Database generation and website implementation

The steps leading to the implementation of the US-SOMO-AF database are outlined in “Methods” section and fully described in the Supplementary Methods sections. Briefly, each entry in the entire AF-v1 (and subsequently -v2) databases was first checked against the corresponding entry in the UniProt database to find the (putative) initiator, signal, and transit peptide regions, which were then removed from the AF PDB files. If propeptide sequence(s) were present, additional PDB files were generated with this/these region(s) removed. If more than a single propeptide was present, permuted structures were generated. These extra AF-derived PDB files have “-pp#” appended to the filename (where “#” is a number). Potential disulfides were identified (allowing a better evaluation of the partial specific volume ${\overline{v}}$ and of *M*) and written as SSBOND records in the curated PDBs, together with HELIX and SHEET information identified using the DSSP^29^ implementation in UCSF Chimera^30^. Batch-mode US-SOMO was used to calculate *M*, ${\overline{v}}$, ${D_{t(20,w)}^{0}}$, ${s_{{\left( {{20},w} \right)}}^{{0}} }$, the derived Stokes’ radius *R*_*s*_, [*η*], *R*_*g*_, the maximum extensions along the principal *X*, *Y* and *Z* axes of the molecule, and the generation of the *p*(*r*) vs. *r* distributions (normalized by the *M* of the structure). SESCA^20^ was used to generate 170–270 nm CD spectra.

**Figure 1:**
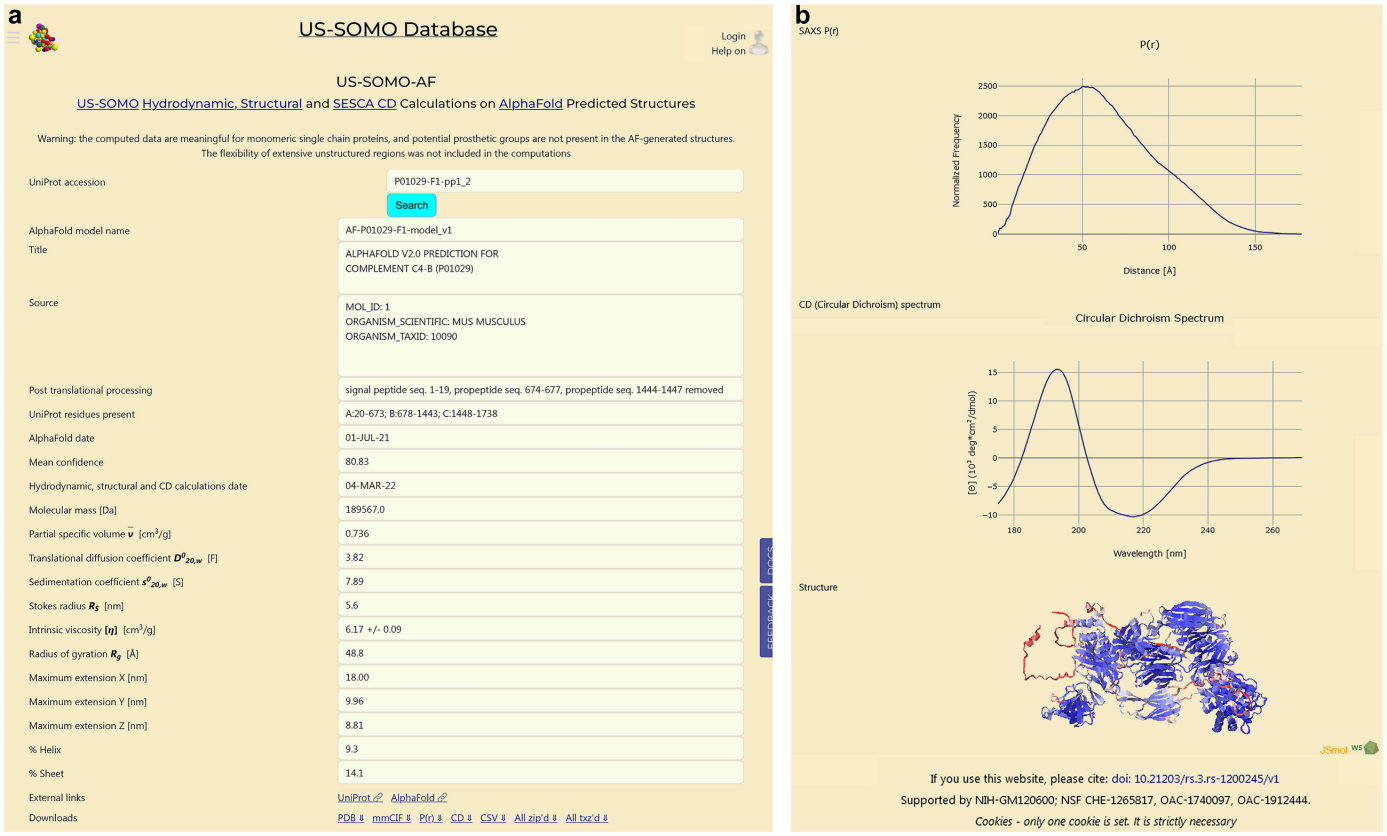
Screenshots of the US-SOMO-AF webpage. Shown are the results for AF-P01029-F1 that includes the removal of the signal sequence and two propeptides. (**a**) The upper part containing text/data information. (**b**) The bottom part showing the computed *p*(*r*) vs. *r* distribution and CD spectrum graphs, and the JSmol representation of the structure.

In Fig. 1a,b, two screenshots of the US-SOMO-AF webpage (https://somo.genapp.rocks) are shown, with panel a featuring the text/data part and panel b containing the graphic output. The header contains hyperlinks to the US-SOMO, SESCA, and AlphaFold websites, and to Ref.^24^. It is followed by a warning message concerning the meaningfulness of the calculations when applied to “real” proteins (see “Discussion” section). By hovering the mouse over entries, an explanation will appear below the corresponding field on the right column. A UniProt accession number, or some initial part of it, can be entered in the first field (typing just the initial part and clicking “Search” will provide a list of corresponding entries, including alternate structures). In any case, if the code is present in the database, the corresponding entry will be shown in the “AlphaFold model name” field, followed by the “Title” and “Source” fields as retrieved from the PDB file. If an initiator methionine, a signal or a transit peptide, and/or any propeptide(s) were identified and their atoms removed from the current AF PDB file, their identity and the stretch of residues involved will be listed in the “Post Translational Processing” field, otherwise “none” will appear. The actual residue(s) stretch(es) present in the structure are displayed in the “UniProt residues present” field. Note that if any propeptide stretch was removed from the middle of a structure, the subsequent chain part(s) was/were renamed “B”, “C”…, without renumbering (see Supplementary Methods for details). The dates on which the AF predictions and US-SOMO/SESCA computations were done appear in their corresponding fields, and in between the “Mean confidence” field reports the calculated mean % per-residue confidence, based on the values present in the AF original PDB file.

The ten fields that follow report the US-SOMO computed parameters. Since the hydrodynamic parameters were computed with the statistically-based ZENO method^31–33^, standard deviations (SD) can be generated. However, a SD is reported only for [*η*], as they are tiny for all other parameters. Note that a calculated ${\overline{v}}$ is provided because besides being used to compute ${s_{{\left( {{20},w} \right)}}^{{0}} }$ from ${D_{t(20,w)}^{0}}$ and *M*, it could also be used to compute an experimental *M* from SAXS data^18^. The bottom two entries report the per-residue % of α-helix and β-sheet as calculated from HELIX and SHEET fields in the curated PDB. They could be compared with CD-derived values, besides comparing experimental and calculated spectra (see below).

External links for the current entry to both UniProt and AlphaFold websites are placed after the parameters listings. Curated PDB- and mmCIF-formatted files for the entry can be retrieved from the provided hyperlinks, as well as text files with the *p*(*r*) vs. *r* distribution and CD spectrum, and a csv-formatted file containing all the identifying information and the single-value parameters. All these files can be also retrieved as single compressed files (zip or tar.xz). Below these hyperlinks, the computed *p*(*r*) vs. *r* distribution and CD spectrum graphs are presented, followed by a JSmol (https://sourceforge.net/projects/jsmol) representation of the structure (see Fig. 1b).

Controls for the visualization and copying as an image of both graphs are provided. JSmol commands are also available to change the representation and export it. The default representation colors the structure according to the per-residue confidence level (red, lowest; blue, highest), but for a more in-depth analysis we refer the user to the original AF website.

In the end, parameters for a total of 365,198 and 1,002,038 structures were generated from the AF-v1 and -v2 databases, respectively (sequences with multiple predicted segments were not included, as the computations of their parameters are meaningless). The AF-v2 structures, including replacements for all AF-v1 structures, are stored in the freely accessible US-SOMO-AF database.

### General data analyses

Although it is beyond the scope of this work to provide extensive data analyses and interpretations, some observations can be made. To begin with, we have randomly selected from the 365,198 AF-v1 curated structures originally present in the US-SOMO-AF-v1 database, a subset containing 41,200 predicted structures with no counterparts in the RCSB PDB^34^ database (https://www.rcsb.org), and we have analyzed their calculated properties (data provided as a spreadsheet, Supplementary Data 1).

**Figure 2:**
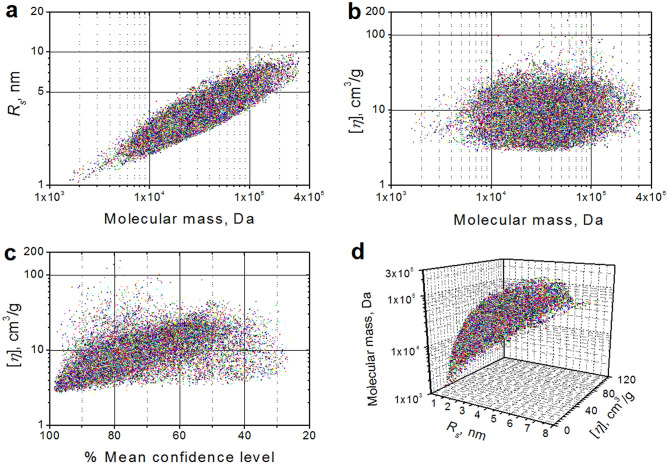
Plots of selected calculated parameters for 41,200 AF-v1 predicted structures with no corresponding entries in the solved structures PDB database. (**a**) *R*_*s*_ vs. *M*, log–log scale. (**b**) [*η*] vs. *M*, log–log scale. (**c**) [*η*] vs. % decreasing mean confidence level, log-lin scale. (**d**) A 3D plot where *M* (log scale) is on the vertical *Z*-axis, and *R*_*s*_ and [*η*] are on the horizontal *X*- and *Y*-axes, respectively (both linear scales).

The graphs in Fig. 2 qualitatively illustrate the potential of selected calculated parameters to distinguish between structures, by observing the spread of the *R*_*s*_ (Fig. 2a) and [*η*] (Fig. 2b) values for a given *M* value (*R*_*s*_ was chosen as a proxy for either ${s_{{\left( {{20},w} \right)}}^{{0}} }$ or ${D_{t(20,w)}^{0}}$, the experimentally determined parameters). It is evident that *R*_*s*_ alone (Fig. 2a) can already distinguish between structures, and its ability to discriminate, albeit somewhat limited, does not substantially change on increasing *M* in the interval 10^4^–10^5^ Da. A significantly larger spread is instead displayed by [*η*], almost independently of *M* (Fig. 2b). To provide a measure of the discriminating ability of *R*_*s*_ and [*η*], we have grouped their values in bins spanning *M* intervals of 5 kDa, and we have computed the pair-wise% difference between each entry. Then, we calculated the percentage of pairs whose % difference was above two pre-established cut-offs, 6% and 9%, reflecting the potential experimental errors conservatively estimated around 3% (see Supplementary Methods). The results are presented in Fig. S1, and clearly show that the discriminating ability is practically constant, independent of both the *M* values and of the number of pairs. For *R*_*s*_ even a 9% cut-off would allow about 70% of the pairs to be discriminated, while for [*η*] this figure is around 90%. [*η*] is, however, more affected by potentially flexible regions not properly taken into account by the computations, sometimes leading to suspiciously very high values. Indeed, a correlation between increasing [*η*] values vs. a decreasing % confidence level in the structure prediction can be seen in Fig. 2c, becoming, however, much less defined when the confidence level goes below 50%. In Fig. S2a, we report a *Z*-scores analysis of this behavior. The shape somewhat follows the number of structures in each bin (Fig. S2b), as expected, since the chances of a sample including an individual from the tail of the distribution increases with sample size. However, the Z-scores seem to flatten out when the confidence level drops below 50%, likely reflecting the lack of [*η*] value clustering. Finally, Fig. 2d shows in 3D how combining two parameters, *R*_*s*_ and [*η*], can effectively increase the ability to discriminate. Another important parameter is *R*_*g*_, but it can rarely be determined by MALS techniques, that have a lower detection limit of ~ 10–11 nm. While SAXS can determine *R*_*g*_, it can also be used to derive the *p*(*r*) vs. *r* distribution^18^, which contains more information and can be directly compared with the one computed from structure. Note that the effect of not taking into account the hydration water in the computation of the *p*(*r*) vs. *r* distribution is relatively minor, and its importance decreases as *M* increases. Therefore, plots involving *R*_*g*_ are not presented here, but could be easily generated from the Supplementary Data 1 spreadsheet.

Since the AF prediction algorithm was trained on the RCSB PDB structures, the exclusion in the above analysis of AF-predicted structures having a counterpart in the RCSB was done to avoid biasing this subset with potentially “correct” calculated parameters. However, it could be also interesting to compare some experimental hydrodynamic parameters with those calculated from both AF-predicted and experimental structures. Unfortunately, in the AF-v1 database there were very few instances that matched the necessary criteria, that is, i. RCSB PDB complete structures of single chain proteins from the same organism also present in the AF-v1 database; ii. without prosthetic groups; iii. having verified sound experimental hydrodynamic parameters, in particular ${D_{t(20,w)}^{0}}$ and ${s_{{\left( {{20},w} \right)}}^{{0}} }$. After perusing Table 2 of Ref.^24^, only three proteins met these criteria, and the comparisons are presented in Table 1. For two proteins, human carbonic anhydrase and human serum albumin, both AF-predicted and PDB structures produced very similar ${D_{t(20,w)}^{0}}$ and ${s_{{\left( {{20},w} \right)}}^{{0}} }$ values (inter-difference of ~ 0.6–0.9%), with excellent matches with ${D_{t(20,w)}^{0}}$ (− 2.4 to + 1.8%) and somewhat worse with ${s_{{\left( {{20},w} \right)}}^{{0}} }$ (− 3.7 to + 5.4%). Notably, a large inter-difference instead was present for soybean trypsin inhibitor (STI; − 4.6 to 4.8%), with the experimental ${D_{t(20,w)}^{0}}$ matched better by the AF prediction and ${s_{{\left( {{20},w} \right)}}^{{0}} }$ by the PDB structure (this apparently odd fact can be explained by either experimental value being potentially incorrect). The inter-difference could be rationalized by superimposing the structures and calculating the RMSD between them, as reported in Table 1. The smallest protein, STI, has the largest RMSD, and this is apparently sufficient to be reflected in the different calculated ${D_{t(20,w)}^{0}}$ and ${s_{{\left( {{20},w} \right)}}^{{0}} }$ values.

**Table 1:** Comparison between experimental and calculated ${D_{{t({20},w)}}^{{0}} }$ and ${s_{{({20},w)}}^{{0}} }$ for three proteins having a crystallographic structure and a predicted AF-v1 structure.

Parameter	Experimental	1AVU.PDB (completed)	% diff. with expt	AF-P01070 (no propeptide)	% diff. with expt	% diff PDB-AF
**Soybean trypsin inhibitor (MW 20,083 g/mol; RMSD between structures 1.72 Å)**						
${D_{{t({20},w)}}^{{0}} }$, F	9.47 ± 0.18	9.91	+ 4.65	9.43	− 0.42	− 4.84
${s_{{({20},w)}}^{{0}} }$, S	2.29 ± n.a.	2.18	− 4.80	2.08	− 9.17	− 4.59

Parameter	Experimental	2CAB.PDB (completed)	% diff. with expt	AF-P00915 (no Met 1)	% diff. with expt	% diff PDB-AF
**Human carbonic anhydrase B (MW 28,744 g/mol; RMSD between structures 0.75 Å)** ^**a**^						
${D_{{t({20},w)}}^{{0}} }$, F	8.89 ± 0.03	9.05	+ 1.80	9.00	+ 1.24	− 0.55
${s_{{({20},w)}}^{{0}} }$, S	3.01 ± 0.19	2.92	− 2.99	2.90	− 3.65	− 0.68

Parameter	Experimental	1AO6.PDB (completed)	% diff. with expt.	AF-P02768 (no propeptide)	% diff. with expt.	% diff PDB-AF
**Human serum albumin (MW 66,437 g/mol; RMSD between structures 1.36 Å)**						
${D_{{t({20},w)}}^{{0}} }$, F	6.31 ± 0.09	6.16	− 2.38	6.21	− 1.58	+ 0.81
${s_{{({20},w)}}^{{0}} }$, S	4.28 ± 0.04	4.47	+ 4.44	4.51	+ 5.37	+ 0.89

The PDB entries had a few missing residues, which were previously manually added^21^; the experimental parameters for all proteins were taken from Ref.^24^.

^a^The 2CAB.PDB entry and the AF-P00915 structure differ at one amino acid position, and have also a position-swap on another two residues; the reported MW is that of the PDB entry.

### Selected examples

**Figure 3:**
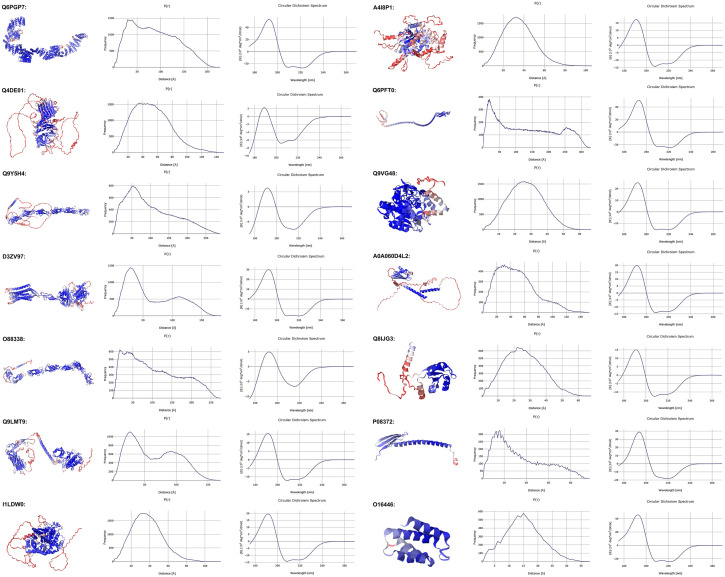
JSmol snapshots of the structures for the entries reported in Table, together with the calculated *p*(*r*) vs. *r* and CD plots.

In Table 2, we have listed 14 entries chosen from the 41,200 mentioned above. They were initially selected to represent intervals from 2.2 to 0.66 in the computed *R*_*g*_/*R*_*s*_ ratio indicating deviation from globular shape (*R*_*g*_/*R*_*s*_ ~ 0.7 for a sphere). A suitable range of [*η*] values was also sought, as well as a good representation of the organisms present in the AF-v1 databases, the presence or absence of a signal peptide, and some spread in the mean % confidence. *M*, *R*_*g*_, *R*_*s*_, and [*η*] were chosen as the calculated parameters, and the entries are ordered by decreasing *M*. Connected to Table 2 is Fig. 3, that displays snapshots of the 3D structures for each entry colored according to the per-residue confidence level, followed by the *p*(*r*) vs. *r* and CD plots.

**Table 2:** Some calculated parameters for a selection of AF-v1 predicted structures with no RCSB PDB counterparts, ordered by decreasing molecular mass.

UniProt accession	Organism	Mean AF % conf.	Signal peptide	Molecular mass [Da]	*R*_*g*_ [nm]	*R*_*s*_ [nm]	[*η*] [cm^3^/g]	Helix%	Sheet%
Q6PGP7^a^	*H. sapiens*	86.48	n/a	175,523	6.98	6.30	10.4	74.5	0.5
Q4DE01^b^	*T. cruzi*	65.88	n/a	102,098	3.99	5.74	12.0	6.5	23.2
Q9Y5H4^c^	*H. sapiens*	75.64	1–28	98,141	8.42	6.56	23.3	9.2	25.5
D3ZV97^d^	*R. norvegicus*	82.81	1–20	94,123	5.55	4.76	8.93	42.8	11.2
O88338^e^	*M. musculus*	84.24	1–21	87,414	8.69	5.87	21.2	5.7	32.5
Q9LMT9^f^	*A. thaliana*	78.02	1–26	82,090	5.16	4.66	8.96	25.4	15.2
I1LDW0^g^	*Glycine max*	75.28	n/a	73,181	2.86	4.16	6.33	32.6	8.9
A4I8P1^h^	*L. infantum*	60.77	n/a	64,586	2.66	3.72	5.18	29.2	9.2
Q6PFT0^i^	*Danio rerio*	81.28	n/a	46,965	11.3	5.75	47.8	66.0	10.8
Q9VG48^j^	*D. melanog*	88.50	1–18	44,673	2.04	2.89	3.44	38.0	9.0
A0A060D4L2^k^	*Zea mays*	68.46	n/a	30,921	3.90	4.06	15.7	32.2	10.4
Q8IJG3^l^	*P. falciparum*	69.83	n/a	19,460	2.05	2.57	5.66	26.4	11.9
P08372^m^	*E. coli*	82.28	n/a	12,010	2.89	2.48	10.5	44.3	21.7
O16446^n^	*C. elegans*	88.31	1–19	8483	1.18	1.66	3.46	68.9	0.0

The corresponding structures and calculated *p*(*r*) vs. *r* distributions and CD spectra can be seen in Fig. 3.

^a^Tetratricopeptide repeat protein 37.

^b^Trans-sialidase, putative.

^c^Protocadherin gamma-a1.

^d^Vomeronasal 2 receptor, 50.

^e^Cadherin-16.

^f^Putative wall-associated receptor kinase-like 13.

^g^Aminotran_5 domain-containing protein.

^h^Adenosine deaminase-like protein.

^i^Flotillin.

^j^Lipase.

^k^BHLH transcription factor.

^l^RNA-binding protein, putative.

^m^Prepilin peptidase-dependent protein C.

^n^Uncharacterized protein.

Table 2 and Fig. 3 provide an insightful glimpse on the great variety of predicted structures and their associated calculated parameters, suggesting that performing some of these checks can indeed boost, or question, their reliability. As expected, CD spectra display differences between most structures, and they are a robust check on the predicted secondary structure content. The variability in [*η*] values in Table 2 appears to confirm its discriminating ability above that of *R*_*s*_, but clearly it is the *p*(*r*) vs. *r* distribution that would provide the best test, although it is the least rapidly experimentally accessible parameter among those considered.

### Comparisons with experimental SAXS-derived data

**Figure 4:**
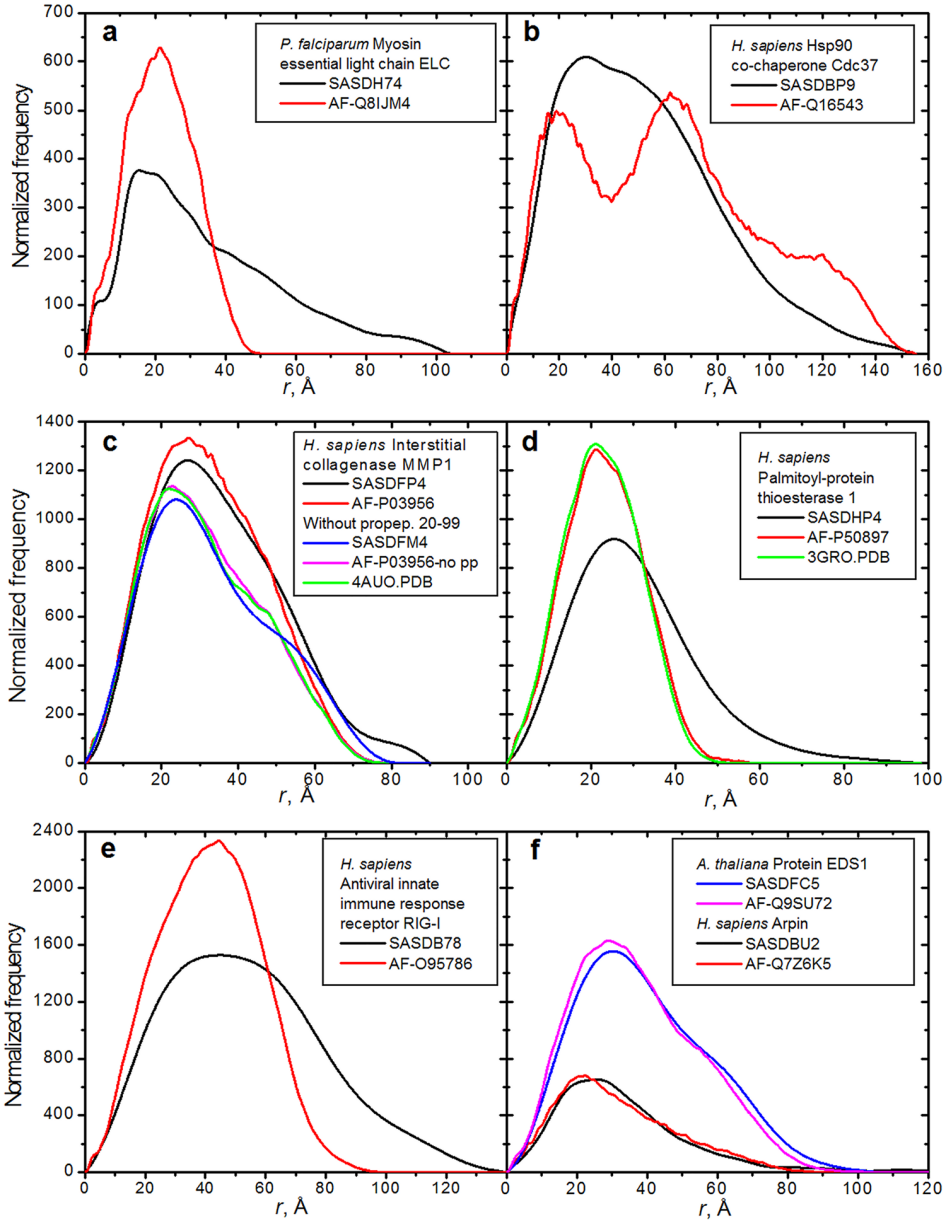
*P*(*r*) vs. *r* curves SAXS-derived and calculated from AF and RCSB PDB structures. (**a**–**f**) Protein source and names, SASBDB, AF (UniProt) and RCSB PDB accession numbers for each entry are indicated in the boxes within each panel. In all panels the experimentally-derived and the AF-calculated *p*(*r*) vs. *r* are black and red lines, respectively. Additional SAXS-derived and AF-calculated *p*(*r*) vs. *r* present in (**c,f**) are blue and magenta lines, respectively. Additional PDB-calculated *p(r*) vs. *r* (green lines) are present in (**c,d**).

To strengthen our case, we have conducted a direct comparison between experimentally-derived *p*(*r*) vs. *r*, retrieved from the SASBDB database^35^ (https://www.sasbdb.org/), and those calculated for the corresponding AF-v1 structures. After a SASBDB search for UniProt codes also present in the AF-v1 database, 473 matching datasets were retrieved. Again, the selection among them was based on the experimental sample being complete, monomeric, and without prosthetic groups, leading to 45 candidates. The final chosen data are presented in Fig. 4, and cover a molecular mass range from 16 to 107 kDa, collected at several SAXS beamlines in either batch or SEC-SAXS mode. In two cases, the corresponding PDB structures were also available. In Fig. 4a, we see a large difference between the SEC-SAXS experimentally-derived *p*(*r*) vs. *r* for the 16 kDa *P. falciparum* myosin essential light chain^36^ (black) and that calculated for the AF-Q8IJM4 structure (red), clearly indicating a more extended conformation in solution. In Fig. 4b, the batch-SAXS experimentally-derived *p*(*r*) vs. *r* for the 44 kDa *H. sapiens* Hsp90 co-chaperone Cdc37 protein^37^ (black) is significantly different from that calculated from the AF-Q16543 predicted structure (red), hinting at a different domains arrangement in solution. The effect of removing the propeptide segment from an AF predicted structure can be appreciated in Fig. 4c, where the 54 kDa *H. sapiens* pro-matrix metalloproteinase-1 (MMP-1) studied before (black) and after (blue) propeptide 20–99 segment cleavage (SEC-SAXS unpublished data collected by R. Holland at Diamond, UK) is compared with the corresponding curated AF-P03956 structures (red and magenta, respectively), and with the *p*(*r*) vs. *r* calculated from chain A in the RCSB PDB structure 4AUO^38^ (green). Here the effect of removing the 9.2 kDa propeptide is noticeable, and subtle differences also appear between the SAXS-derived and calculated *p*(*r*) vs. *r*, with the AF-P50897 and PDB-derived structures being almost identical. Another complete accordance between AF- and PDB-derived (AF-P50897 and 3GRO, unpublished) *p*(*r*) vs. *r* can be seen in Fig. 4d for the 31 kDa *H. sapiens* palmitoyl-protein thioesterase 1 (PPT1; red and green, respectively), but both are quite different from the SEC-SAXS derived data^39^ (black) that point to a more elongated structure. A noticeably more elongated structure is also apparent in Fig. 4e by comparing the SEC-SAXS derived^40^ *p*(*r*) vs. *r* for the 107 kDa *H. sapiens* probable ATP-dependent RNA helicase DDX58 (black) with the one calculated for AF-O95786 (red). Finally, in Fig. 4f are two cases where SEC-SAXS-derived and AF-calculated *p*(*r*) vs. *r* yield nearly identical curves, the 72 kDa *A. thaliana* enhanced disease susceptibility 1 (experimental^41^, blue; AF-Q9SU72, magenta) and the 25 kDa *H. sapiens* arpin isoform 1 (experimental^42^, black; AF-Q7Z6K5, red).

### Conformational variability

**Figure 5:**
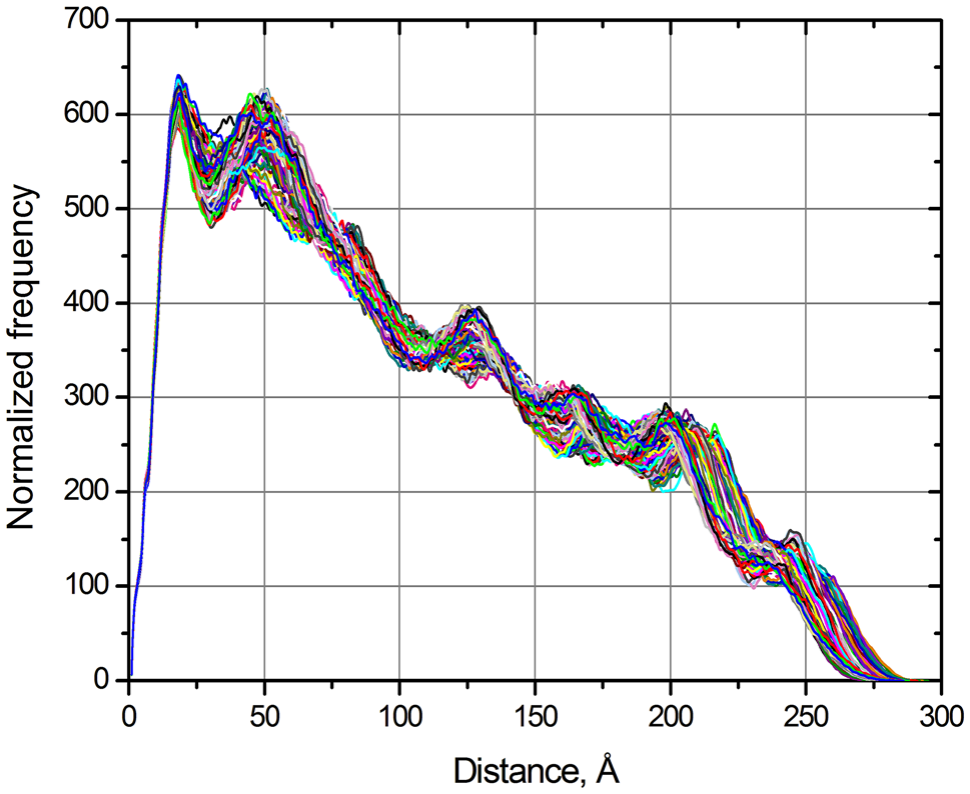
Calculated *p*(*r*) vs. *r* distributions for the 100 conformations generated in the DMD run on the AF-predicted O88338 structure.

To provide an additional test of the discriminatory ability of the hydrodynamic parameters and *p*(*r*) vs. *r* distribution, we have selected the O88338 Cadherin-16 from *M. musculus* structure (see Table 2, Fig. 3) that contains a number of independently folded domains connected by linkers, and we have run a Discrete Molecular Dynamics (DMD)^43,44^ simulation to expand its conformational space (see Supplementary Methods for details), followed by hydrodynamic and *p*(*r*) vs. *r* calculations on 100 produced structures. As can be seen in Supplementary Video 1, sufficiently different alternative conformations were generated within an overall frame, allowing an evaluation of the spread in the predicted parameters and their potential discriminating capability. For instance, the *R*_*s*_ spread, 5.88–6.16 nm (a ~ 4.5% change) would be barely above experimental error in distinguishing between the most different conformations in this set, while the spread in [*η*], 20.8–23.8 cm^3^/g (a ~ 12.6% change), would clearly allow distinguishing between many conformations (all this set’s individual data are in Supplementary Data 2 spreadsheet, and the *R*_*s*_ and [*η*] are reported in each video frame, along with the *p*(*r*) vs. *r* distributions). Even more striking is the variation in the *p*(*r*) vs. *r* distributions that are also collectively reported in Fig. 5. Thus, even for such a restricted structural variation, comparing experimental and calculated parameters can provide reliable tests of the predicted structures.

### Effects of long unstructured, potentially flexible regions

**Figure 6:**
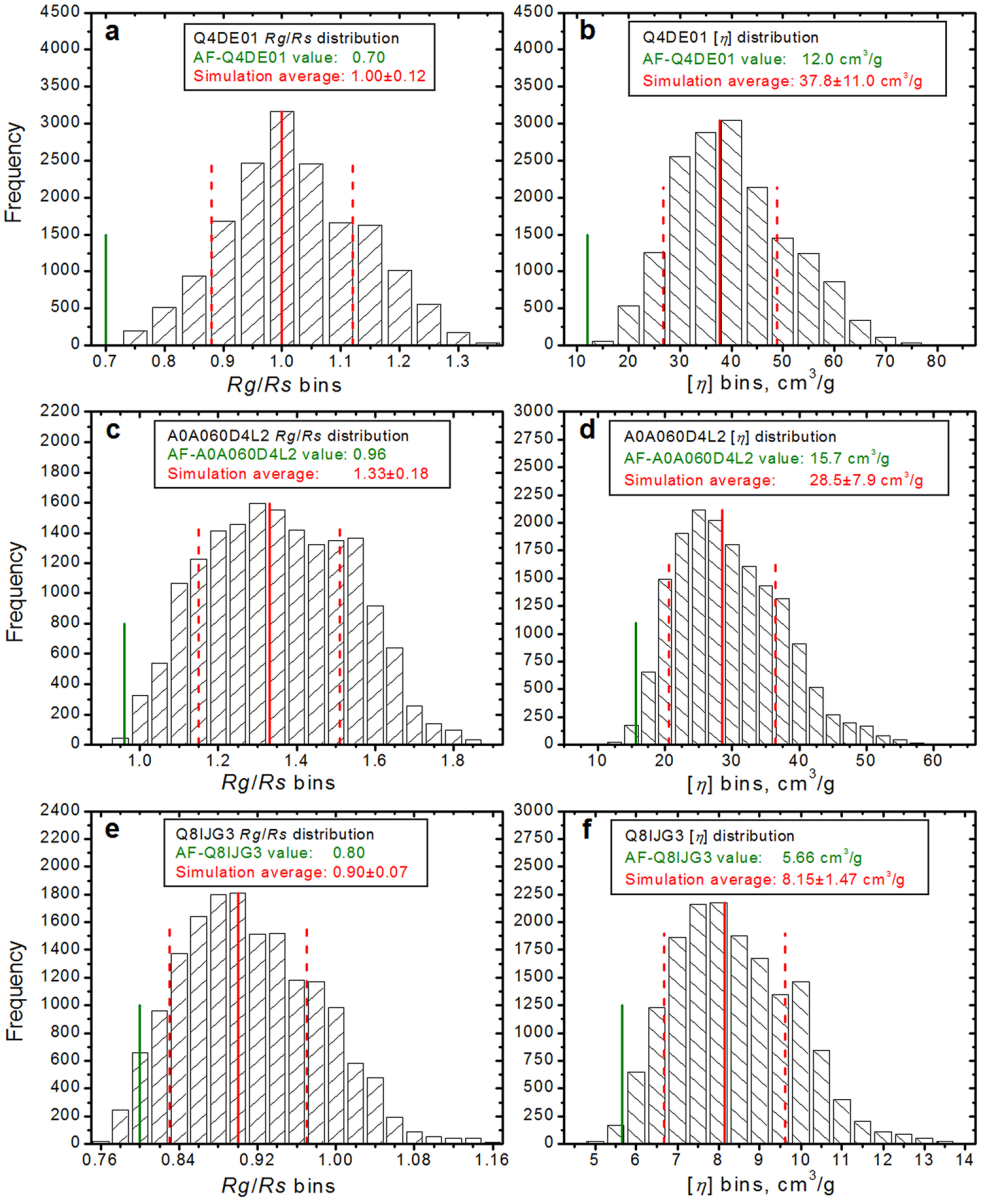
Histograms of the calculated parameters for the MMC-generated conformations of three AF-predicted structures from Table 2. Shown are the distributions of *R*_*g*_/*R*_*s*_ (**a**,**c**,**e**) and of [*η*] (**b**,**d**,**f**) calculated for AF-Q4DE01 (16,520 conformations, (**a,b**)), AF-A0A060D4L2 (16,666 conformations, (**c,d**)), and AF-Q8IJG3 (16,367 conformations, (**e,f**)). In each panel, the vertical green lines mark the location of the starting structure parameters, while the vertical solid and dashed red lines indicate the average ± SD over all conformations (the actual values are reported in each panel’s inside legend).

Finally, we have also explored the effect of generating a large number of conformations for AF-predicted unstructured parts in three of the AF-v1 proteins shown in Table 2 and Fig. 3, AF-Q4DE01 (residues 1–72 and 746–957), AF-A0A060D4L2 (residues 1–118), and AF-Q8IJG3 (residues 1–40), by producing over 16,000 full structures for each entry. Since molecular dynamics or even DMD runs would have been prohibitively time-consuming, we used the Monomer Monte Carlo (MMC) simulation tool in the SASSIE-web suite^45^, followed again by batch-mode US-SOMO to compute the hydrodynamic parameters (see Supplementary Methods for details; an animation of 100 randomly chosen among the generated structures for AF-A0A060D4L2 is presented as Supplementary Video 2). Besides calculating the averages ± SD of each parameter, we also statistically analyzed the data, producing distribution histograms. The results can be seen in Fig. 6, where histograms of the distributions of the calculated *R*_*g*_/*R*_*s*_ ratio (panels a,c,e) and [*η*] (panels b,d,f) are shown. The starting conformations and the average ± SD values are reported in each panel’s internal label, and are marked on the plots as solid green, and solid and dashed red vertical lines, respectively. From these graphs, the noticeable increase and spread of calculated values is evident for both parameters, the latter being reflected in the large SD associated with the average values. Some differences can be seen, with [*η*] more correlated to the size of the unstructured regions (decreasing from panels a,b to panels e,f in Fig. 6) and *R*_*g*_/*R*_*s*_ apparently better able to pick up a bimodal distribution (Fig. 6 panel c). Note that since the MMC simulations we ran did not involve an energy penalty term in accepting/rejecting conformations at each step, but only an overlap check, these calculated average values cannot be directly compared with potential experimentally-derived values, as also indicated by the large associated SDs. Nevertheless, they surely confirm that the conformation of unstructured parts will severely affect the hydrodynamic properties in solution, reinforcing the importance of measuring them.

## Discussion

We have presented here a new database stemming from the AlphaFold predicted protein structures databases. We initially worked with the AF-v1 release, whose entries were utilized for the tests reported here, and we have already extended it to the AF-v2 recent release. The resulting US-SOMO-AF database contains calculated hydrodynamic and structural parameters whose experimental determination should be within the reach of scientists working with a particular protein for which a “hard” structure is either currently unavailable or in the making. Indeed, it is interesting to note that crystallographers and cryo-electron microscopists are already suggesting using AF-predicted structures to solve experimental structures by molecular replacement methods^46^. Performing some rapid tests and comparing the results with those we provide in the US-SOMO-AF database could save them valuable time and perhaps hint at twists that should be applied to a predicted structure to better fit the X-ray, cryo-EM, and NMR data. In this respect, we would like to point out a tool present in the US-SOMO program that allows one to color-code a visualized structure based on the contribution of residues to a particular set of distances in a *p*(*r*) vs. *r* distribution^47^. For instance, this could provide an easier identification of domains that under- or over-contribute to that set of distances. This is another reason why we chose to produce real-space *p*(*r*) vs. *r* distributions instead of reciprocal-space simulated SAXS intensity vs. scattering vector curves, for which a wide variety of methods, often quite computationally intensive, exist^48^. More in-depth analyses could be subsequently performed on case-by-case basis.

For a more general application, assessing the reliability of a predicted structure could lead to better designed function/structure relationship experiments. The availability of the US-SOMO-AF database has the distinctive advantage of allowing a quick comparison without the need to master the expertise necessary to soundly calculate the relevant solution parameters.

There are, of course, a series of drawbacks associated with these computations. First and foremost, all the AF predicted structures consider all proteins as single chain entities. Efforts are underway (see^6^) to cope with this issue by allowing multi-chain predictions, and when an evolution in that sense appears in the AF database (only a general tool is presently available, see https://alphafold.ebi.ac.uk/faq), all parameters could be re-calculated for a new set.

A second evident drawback resides in the post-translational modifications that many proteins undergo. None were considered by the AF team, and we have made an important first step by removing the initiator methionine, signal and transit peptides, and producing alternate structures with/without propeptides. This resulted in about ~ 11% (~ 110,000 over ~ 1,002,000) of the AF-v2 structures being modified by our procedures, a sizeable amount. The remaining most important modification, affecting the calculated parameters, is glycosylation (e.g., see Table 1 in Ref.^49^). While UniProt provides a list of potential glycosylation sites for entries, and publications describing them when available, presently there is no direct way to have the composition of each carbohydrate associated with a particular site. This is a pity, as methods for building complex carbohydrates are already available and/or under development (see^50^), and it should be relatively straightforward to automatically add them at the appropriate sites. Indeed, this has just been independently advocated in a recent letter^51^. Even in absence of time-consuming molecular dynamics minimization steps, this simple addition could increase the reliability of calculated hydrodynamic and structural parameters. While we hope that such an important step will be taken at the UniProt and/or AlphaFold databases level, users that need to refine the calculations on a predicted structure after having manually added any prosthetic group can easily do so by using one of the downloadable (http://somo.aucsolutions.com) US-SOMO versions.

The third drawback is the handling of flexibility, especially if large unstructured parts are predicted. Here the US-SOMO-AF database can only raise red flags, such as very high predicted [*η*] values associated with visualized extended, unstructured parts. Dealing with these issues requires much longer calculations involving either Monte Carlo methods or Brownian dynamics simulations (see^52^), that would require a major effort to be applied systematically on > 1,000,000 structures. While our simple test with three proteins (Fig. 6) just shows the complexity of the problem, comparing some experimental parameter with those calculated on a starting AF structure would still be quite informative.

All current data has been also deposited to Dryad (https://datadryad.org), which promises preservation. We expect to maintain the website as long as computational resources are available and community interest continues. The website leverages a framework (see Supplementary Methods) which is actively maintained, greatly simplifies website maintenance and updates, and is being used by multiple projects, some since 2013. Our plan is to update the database as new AlphaFoldDB datasets are released. However, modifications or additions to AlphaFoldDB released datasets (e.g., inclusion of carbohydrates), may require us to seek additional funding and/or solicit community contributions to enhance our processing pipeline and/or its component programs (hydrodynamic, structural and spectra calculations). We welcome any group or individual that wishes to host or contribute to the website, database or processing pipeline. They can contact us through the feedback tab of the website or email us directly.

All considered, we believe that the publicly available (https://somo.genapp.rocks) US-SOMO-AF database described here will become a useful tool allowing the research community, by comparing one or more experimentally-determined parameters with the corresponding computed ones, to quickly evaluate the compatibility in solution of an AlphaFold predicted protein structure.

## Methods

Production of the results presented in this paper required five major steps: collect the AlphaFold entries and additional metadata; prepare the structures for hydrodynamic, structural and CD calculations; compute the hydrodynamic, structural and CD properties; build a database containing the hydrodynamic properties and additional metadata; and finally build a website allowing users convenient access to the database.

After downloading the AlphaFold-v1 and -v2 databases, we prepared the structures by removing the post-translational processing regions, where present, identified from the UniProt website. We utilized US-SOMO^23–25^ to compute hydrodynamic and structural properties. The US-SOMO suite uses a bead modeling strategy which takes into account the theoretical amount of “bound” hydration water, and the ZENO computational algorithm^31–33^ was employed to calculate the hydrodynamic parameters in a rigid-body frame. US-SOMO was also used to compute the *p*(*r*) vs. *r* distribution on not-hydrated structures, using SAXS-related parameters. To compute the CD spectra, we used SESCA^20^.

All the computed results were collected and inserted into a database. Full descriptions for all these steps can be found in the Supplementary Methods section.

## Supplementary Information

Supplementary Information 1.

Supplementary Video 1.

Supplementary Video 2.

Supplementary Information 2.

Supplementary Information 3.

## Data Availability

The datasets generated and/or analyzed during the current study are available in the US-SOMO-AF website, https://somo.genapp.rocks. All computed data has been deposited in a public data repository: https://doi.org/10.5061/dryad.jq2bvq89s.
