# Supplementary material for: A database of calculated solution parameters for the AlphaFold predicted protein structures
Source: Sci Rep. 2022 May 5;12:7349. doi: 10.1038/s41598-022-10607-z (PMC9072687; doi:10.1038/s41598-022-10607-z)
Supplement: Supplementary file 1 — Supplementary Information 1. [file 41598_2022_10607_MOESM1_ESM.pdf]

A database of calculated solution parameters for the AlphaFold predicted protein structures

Emre Brookes[1] and Mattia Rocco[2]

[1]Department of Chemistry and Biochemistry, The University of Montana, 32 Campus Dr, Missoula, MT 59812, USA. Email: emre.brookes@umontana.edu

[2]Proteomica e Spettrometria di Massa, IRCCS Ospedale Policlinico San Martino, Largo R. Benzi 10, I-16132 Genova, Italy (retired, official visitor). Email: mattia.rocco@quipo.it

Supplementary Information

Supplementary Figures

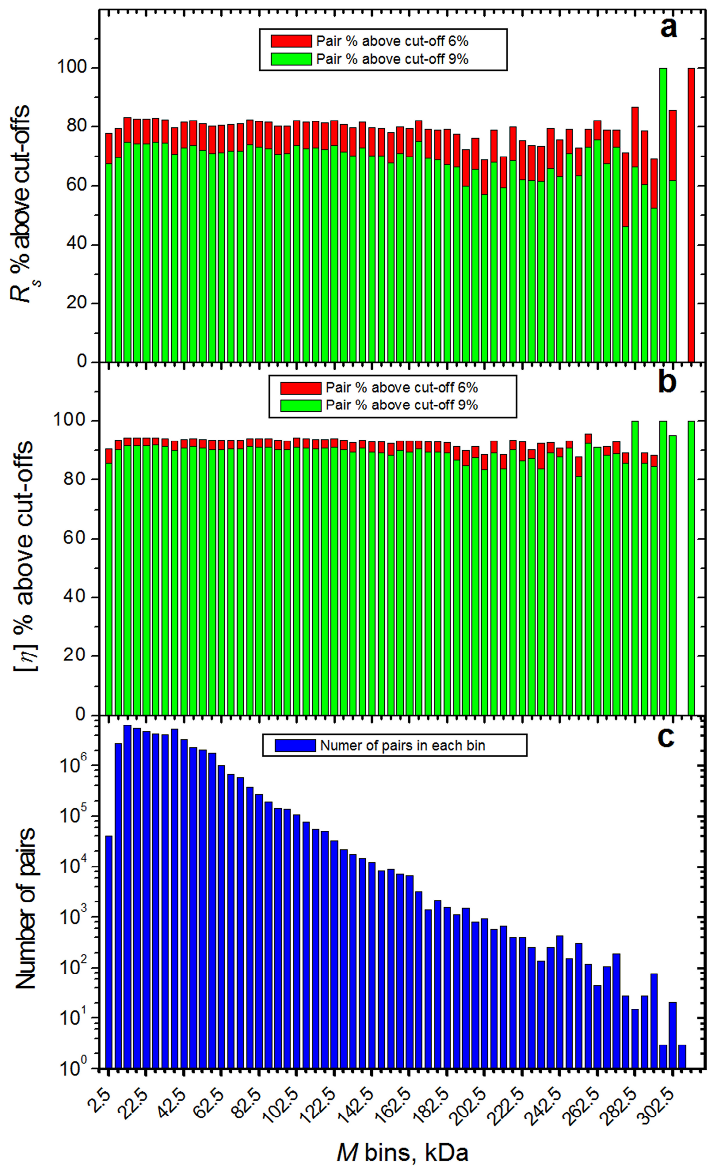

Figure S1 - Histograms of the pair-wise analyses for the R_s and [η] values in the Supplementary Data 1 spreadsheet, in M bins of 5 kDa increments. Panel **a**, % of pairs of R_s values whose % difference is above two specified cut-offs, 6 % (red bars) and 9 % (green bars). Panel **b**, % of pairs of [η] values whose % difference is above two specified cut-offs, 6 % (red bars) and 9 % (green bars). Panel **c**, number of pairs in each M bin (note that the x-axis values shown refer to the center of each bin). See Supplementary Methods for computational details.

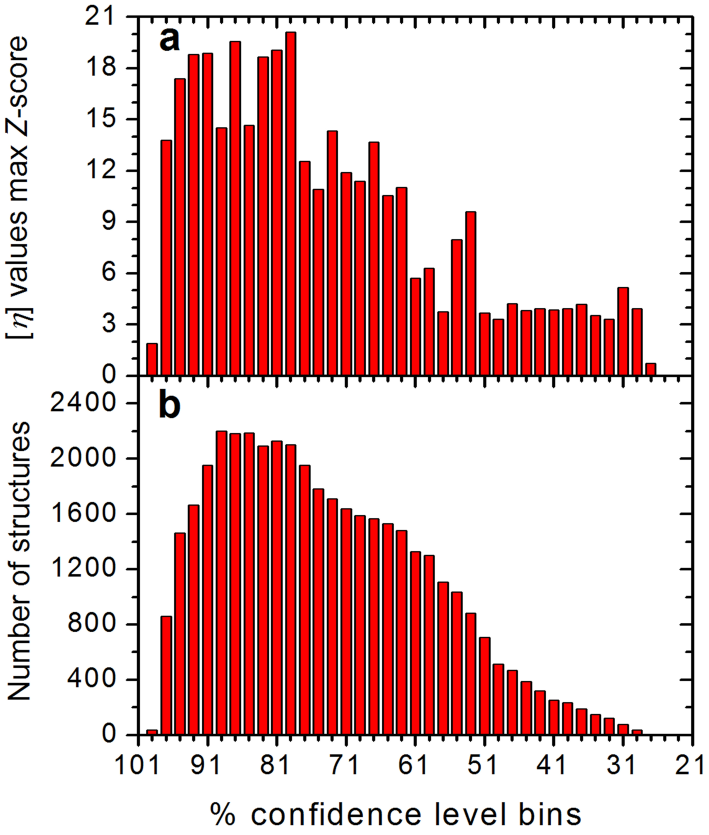

Figure S2 -Histograms of the maximum *Z*-scores of [η] values (panel **a**) and number of structures (panel **b**) in bins of 2% confidence level (see Fig. 2**c**). For each bin, a normal distribution was computed and the maximum *Z*-score was found (see Supplementary Methods). Note that the *x*-axis values shown refer to the center of each bin.

Supplementary Data Legends

Supplementary Data 1 - Excel spreadsheet containing a subset of 41,200 predicted structures randomly selected from the AF-v1 database with no counterparts in the RCSB PDB, with their calculated properties.

Supplementary Video 1 - Animation showing the 100 DMD-generated structures for the O88338 Cadherin-16 from *M. musculus*, with their calculated R_s and $[\eta]$ and the $p(r)$ vs. r distributions reported in each video frame.

Supplementary Data 2 - Excel spreadsheet containing the calculated parameters for the 100 DMD-generated structures of the O88338 Cadherin-16 from *M. musculus*.

Supplementary Video 2 - Animation of 100 structures randomly selected from the >16,000 generated by an MMC run on the AF-A0A060D4L2 entry, keeping static residues 119-290.

Supplementary Methods

US-SOMO-AF database and data organization background

We chose a NoSQL MongoDB (https://www.Mongodb.com) database to store the hydrodynamic calculations and metadata due to its familiarity to the authors and its native support by the chosen website framework. Structural and CD properties were kept as separate files to simplify website direct download access. All steps processing large numbers of structures were performed by creating and running command line scripts. All work was done on the PDB files and not the mmCIF files, as we knew the hydrodynamic, structural and CD calculation programs we utilized supported the PDB format.

Collecting and pre-processing the AF database entries

The first step was to download (http://ftp.ebi.ac.uk/pub/databases/alphafold) the entire AlphaFold (AF) database. We initially processed the AF-v1 data with a collection of scripts. We subsequently built a pipeline (https://github.com/ehb54/somoaf-pipeline) to process the data, which will be outlined here. The pipeline is written in Perl (https://www.perl.org) and consists of six stages. Note the pipeline can be run in parallel by providing unique lists accession codes to each job. Initially a configuration script is edited (config.json) from the template (config.json.template) with appropriate paths set. Each stage takes as input a list of UniProt accession codes.

Pipeline processing stage 1 - collect data

The first pipeline stage, collect.pl, collects the FASTA sequence and UniProt Features from UniProt (the latter from the UniProt REST API, https://www.uniprot.org/help/programmatic_access) and writes these into output files used by the next pipeline stage.

Pipeline processing stage 2 - build sequence summary information

The second stage, chains.pl, examines the AF PDB, FASTA and UniProt Features and builds a chains file for each accession code for consumption by the subsequent stage. The UniProt Features of category MOLECULE_PROCESSING have types of “init_met”, “signal”, “propep”, “peptide” and “chain”. This information is manipulated to produce the chains file. From these Features, the stage makes multiple handling decisions. Unknown start and end residue sequences (represented as

a question mark on the UniProt website and a tilde in the UniProt Features) are managed by joining two Features if the prior Feature's end and the subsequent Feature's start are both unknown. If only one Feature start or end is unknown, then the unknown value is set to the known value appropriately incremented or decremented. Gaps in Features are managed by joining if they are the same type or extending the "chain" if they are different types of Features and one is a "chain". Feature overlaps are managed by cutting features to remove the overlap, giving precedence to chains. Features may contain multiple isoforms, in which case we only consider the first encountered. The produced chains file is in a human-readable format consisting of the components of the structure (*e.g.* init_met 1 1, transit 2 20, chain 21 223, propep 224 244). Additional files are created at this stage which contain detailed processing notes and any errors encountered (*e.g.* mismatch between FASTA and AF PDB residue count). At this stage, accession codes with multiple AF frames are dropped, as these are not useful for further computations.

Pipeline processing stage 3 - initial PDB and PDB variant creation

The third stage, pdbs.pl, reads the accession's chains file and cuts any initial methionine, transit and signal peptides. This becomes one output PDB. For any propeptides present, all permutations of removal are computed and variant output PDBs are created, each noted with a -pp# extension. For example, if the first and third propeptide are removed, the extension will be -pp1_3. Note that when removing propeptides causes a sequential residue number break, unique chain ids are assigned, (*e.g.* B, C), as US-SOMO's treats broken chains (residues missing) differently than intact chains, leading to differences in the bead models generated (normally, the main chain beads are always positioned at each peptide bond center of mass, but if breaks are present, the main chain beads are positioned at each CA). Note also that when post-translational modifications would result only in a set of small peptides, no additional PDB structures were generated (*e.g.* Q19165).

Pipeline processing stage 4 - finalize PDBs and mmcifs

The fourth stage, pdb2.pl, takes each PDB produced by the previous stage and finalizes it by adding additional records. For SSBONDs we utilized the disulphide bond identification feature recently developed in US-SOMO[25], and modified the US-SOMO code to expose this feature via the

command line. SEQRES records are updated to reflect the actual residues and chains present in the PDB. To add α-helix and β-sheet information into the PDB files, UCSF-Chimera[30] is used, which includes a DSSP-based algorithm[29] to identify stretches of secondary structure. Chimera adds the HELIX, SHEET and CONECT records to the PDB file. Chimera also uses the updated SEQRES record to add OXTs for cases where chain breaks were introduced or the C-terminal truncated due to the removal of a propeptide. REMARK records are added to the PDB detailing this stage's processing steps and the residues remaining. Next, the stage produces mmCIFs for the website using RCSB's MAXIT software (https://sw-tools.rcsb.org/apps/MAXIT). This required two processing runs as MAXIT needed to first convert the PDB file to a CIF file and subsequently convert the CIF file to an mmCIF file. Finally, an additional PDB is created with the temperature factors replaced by AF's confidence levels mapped to values appropriate for JSmol visualization.

Pipeline processing stage 5 - compute

The fifth stage, compute.pl, computes the hydrodynamic parameters, structural data (SAXS $p(r)$ vs. r) and circular dichroism spectra, and assembles the resulting data. To compute the hydrodynamic parameters and structural properties, the US-SOMO software is called, which includes several modeling options and multiple hydrodynamic parameter calculation algorithms[23-25]. For this work, we chose the "SoMo with overlaps" bead modeling method, which coupled with the ZENO computational method[31-33] has produced the best matching between experimental and computed parameters for an extended set of test proteins[21,24]. The SoMo with overlaps method is based on the original SOMO method in which each main and sidechain segments for every amino acid are represented by a bead whose volume includes that of the theoretically bound hydration waters[53]. Although US-SOMO now includes the state-of-the-art GRPY method[25,54] to compute the hydrodynamic parameters of bead models with overlaps, it is significantly more computationally intensive, and its main advantage would be to produce also the rotational diffusion parameters (such as the rotational correlation time(s)), which are, however, more difficult to accurately measure. All the computations were carried out under standard solvent conditions (water at 20 °C, pH 7).

US-SOMO is also used to compute the SAXS $p(r)$ vs. r curves[47]. To allow execution by the pipeline, the development US-SOMO code was enhanced to support the execution of script files.

To compute the CD spectra from our prepared PDB files, the program SESCA[20] is used. SESCA's SESCA_main.py program is called on our PDB files producing a CD_comp.out file containing the CD spectrum.

Additional database parameters are computed, including the percentage of α-helix and β-sheet residues present in the PDB, and the average AF confidence level. Various database fields such as the PDB's TITLE, SOURCE, and the content of REMARKs previously generated (fourth pipeline stage) are extracted from the PDB. These database parameters, along with the hydrodynamic calculation results, obtained by reading US-SOMO's csv output, are collected into a MongoDB command line script output at this stage. Finally, compressed tar and zip archives are built.

Pipeline processing stage 6 - package

The final stage, package.pl, places all results in defined target directories to be easily uploaded to the website. All MongoDB commands are concatenated into a single file, which is used to update the database on the website.

All the above stages are run in sequence on a provided set of accession codes, making it relatively straightforward, however computationally intensive, to update the database whenever AF provides updated structure predictions. Simultaneous multiple runs of the pipeline are supported, each running on unique sets of accession codes.

Processing performance notes

The most time-consuming step in the processing pipeline is the hydrodynamic calculations which took approximately one minute per PDB on either an AMD EPYC 7742 (University of Lethbridge, shared) or an AMD EPYC 7764 (Texas Advanced Computer Center - TACC, Lonestar 6, exclusive node access). AF-v1 computations were completed exclusively on University of Lethbridge's EPYC in shared mode. However, to gain additional throughput, we enabled the pipeline on TACC's resources. A dedicated TACC dual EPYC node gave us throughput of 100 PDBs per minute, requiring approximately 165 node hours to compute the entire AF-v2 database.

Website implementation

The website was created with the GenApp framework[55]. The website runs in a Docker (https://www.docker.com) container based on Ubuntu 20.04.3 LTS (https://ubuntu.com) with PHP 7.4.3 (https://www.php.net), MongoDB 4.2.17 and Apache 2.4.41 (https://httpd.apache.org). GenApp application development works by creating various definition files and provides a rich user interface including advanced plotting (Plotly, https://plotly.com) and support for atomic structure display (JSmol, https://sourceforge.net/projects/jsmol). The GenApp Docker container was built with the GenApp provided Dockerfile. A JSON (https://www.json.org) formatted module definition file was written detailing all the inputs, outputs, user interface layout and a reference to the underlying executable. GenApp provides limited constraints on the language of the module's executable. PHP was chosen for the module's executable due to its fast startup, good support for JSON and available MongoDB interface. A PHP executable file was created which: *a*-consumes an input object as described in the module definition file; *b*-does the appropriate lookups from the MongoDB, including, if multiple records are found matching the search string, an interactive user refinement to a single result; *c*-populates the output object, as described in the module definition file, with the hydrodynamic parameters, metadata and links to the PDB, mmCIF, CSV, $p(r)$ vs. r, CD, zip and compressed tar files for user download and a reference to the PDB for the JSmol viewer; *d*-and finally outputs the JSON output object. JSON formatted directives and menu files were modified from provided templates to, respectively, specify the overall website details and include a reference to the created module. The GenApp framework engine was run to build the complete website. Refining the website was an iterative procedure consisting of modifying the definition files and/or the module's executable, running the GenApp engine, and testing in a web browser. Website creation was and hosting is done on the NSF supported Jetstream cloud[56] made possible by an XSEDE[57] allocation to E.B.

DMD simulations of AF-O88338

To expand the conformational space of AF-O88338, we used US-SOMO's interface[58,59] to a Discrete Molecular Dynamics (DMD) program[43,44]. Operations were carried out with the Linux

version of US-SOMO operating on a cluster[56,57]. Relaxation was run for 5 ps at 0.7 kcal/mol/k_B, the production was run for 5 ns at 0.6 kcal/mol/k_B. The Andersen thermostat was used for both the relaxation and run stages.

Monte Carlo Molecular Mechanics

To expand the conformational space of Q4DE01, A0A060D4L2 and Q8IJG3, we utilized the Monomer Monte Carlo module of SASSIE-web[45] (https://sassie-web.chem.utk.edu/sassie2/). The flexible regions were provided as residues 1-72 and 746-957, 1-118, 1-40, respectively. The number of trial attempts were set to 20,000. For all other input fields, defaults were used. As a fraction of the structures are rejected due to steric clashes, the final counts of accepted (produced) structures were 16,520, 16,766, 16,367, respectively.

Data analysis

For the pair-wise comparisons between the calculated R_s and $[\eta]$ values for the subset of 41,200 predicted structures in the AF-v1 database, we first estimated the average % SD of the experimental $D^0_{t(20,w)}$ and $s^0_{(20,w)}$ values for the proteins in Table 2 of ref.[24]. For the 21 $D^0_{t(20,w)}$ values and for the 24 $s^0_{(20,w)}$ values with an SD available, the average % SD is 2.3 ± 1.5 % (range 0.2 - 4.1 %, with a 5.8 % outlier), and 1.5 ± 1.2 % (range 0.4 - 2.5 %, with a 6.3 % outlier), respectively. Given these numbers, we conservatively assumed a ±3 % SD as a reasonable upper bound for experimentally-derived R_s values, and this limiting % SD was also assumed for $[\eta]$. The computed parameters were subdivided in M bins of 5 kD increments, and the pair-wise % difference between R_s (or $[\eta]$) values in each bin was computed as $[|(a - b)| / (a+b)/2] \times 100$, where a and b represent R_s (or $[\eta]$) pairs. Finally, we counted in each M bin the number of R_s (or $[\eta]$) pairs having a % difference ≥ 6 or ≥ 9 (twice or three-times the estimated experimental average error, respectively). For the evaluation of the maximum Z-score[60], the computed $[\eta]$ values were placed in 2% increments confidence level bins (from 100% to 20%). For each bin, mean and SD were computed, from which the maximum Z-scores were then derived. All the computations involving binning and subsequent steps were done with custom Perl scripts.

The distribution of calculated hydrodynamic parameters for AF-Q4DE01, AF-A0A060D4L2, and AF-Q8IJG3 following MMC simulations was done with the Histogram function in Microsoft® Office Excel 2003. For each MMC run, the number of bins and their size were selected as a function of the hydrodynamic parameters' spread.

Graphs, figures, and movies preparation

Non-website graphs were prepared with Origin v. 6.0 (Microcal, now OriginLab, https://www.originlab.com). Figures were assembled using PaintShopPro v. 5.3 (JASC Software, now Corel, https://www.paintshoppro.com). The Supplementary Video 1 protein structures images were generated by UCSF Chimera, which was also used to directly produce the Supplementary Video 2. For Supplementary Video 1, the movie's $p(r)$ vs. r distribution plots were generated by Gnuplot (http://www.gnuplot.info). Plots and text were then inserted using ImageMagick (https://imagemagick.org). The final Supplementary Video 1 was assembled with FFmpeg (https://www.ffmpeg.org).

Supplementary references

53. Rai, N. et al. SOMO (SOlution MOdeler) differences between X-Ray- and NMR-derived bead models suggest a role for side chain flexibility in protein hydrodynamics. *Structure* **13,** 723-734 (2005). Doi: https://doi.org/10.1016/j.str.2005.02.012

54. Zuk, P.J., Cichocki, B. & Szymczak, P. GRPY: an accurate bead method for calculation of hydrodynamic properties of rigid biomacromolecules. *Biophys. J.* **115,** 782–800 (2018). Doi: https://doi.org/10.1016/j.bpj.2018.07.015

55. Savelyev, A. & Brookes, E. GenApp: Extensible tool for rapid generation of web and native GUI applications. *Future Gener. Comput. Syst.* **94,** 929-936 (2019). Doi: https://doi.org/10.1016/j.future.2017.09.069

56. Stewart, C.A. et al. Jetstream: a self-provisioned, scalable science and engineering cloud environment. In: *XSEDE '15: Proceedings of the 2015 XSEDE Conference: Scientific*

Advancements Enabled by Enhanced Cyberinfrastructure. Association for Computing Machinery, New York, NY, USA, pp. 1-8 (2015).
Doi: https://doi.org/10.1145/2792745.2792774

57. Towns, J. et al. XSEDE: Accelerating Scientific Discovery. *Comput. Sci. Eng.* **16,** 62-74 (2014). Doi: https://doi.org/10.1109/MCSE.2014.80
58. Brookes, E. et al. US-SOMO cluster methods: year one perspective. In: *XSEDE '13 Proceedings of the conference on extreme science and engineering discovery environment: gateway to discovery*, Article 65, pp 1-2 (2013). Doi: https://doi.org/10.1145/2484762.2484815
59. Rocco, M. & Brookes, E. Dynamical aspects of biomacromolecular multi-resolution modelling using the UltraScan Solution Modeler (US–SOMO) suite. In: *The Future of Dynamic Structural Science. NATO Science for Peace and Security Series A: Chemistry and Biology*. Howard, J., Sparkes, H., Raithby, P. & Churakov, A. (eds). Springer, Dordrecht, pp 189–199 (2014). Doi: https://doi.org/10.1007/978-94-017-8550-1_13
60. Roessner, U., Nahid, A., Chapman, B., Hunter, A. & Bellgard, M. Metabolomics –The Combination of Analytical Biochemistry, Biology, and Informatics. In: Comprehensive Biotechnology (Second Edition). Moo-Young, M. (ed). Academic Press, Cambridge, MA (USA), Chapter 1,33, pp. 447-459 (2011). Doi: https://doi.org/10.1016/B978-0-08-088504-9.00052-0
